# Some Unusual Complications of Measles

**Published:** 1904-06-11

**Authors:** 


### SOME UNUSUAL COMPLICATIONS OF MEASLES.

In quite recent months three cases of measles having very unusual complications or sequelæ have been put on record and it seems desirable these should be widely known. The first, reported by Dr. Dan McKenzie,[1] was that of an infant aged 18 months, who had an attack of measles on March 2nd, 1903. After an uneventful course for three weeks the temperature rose to 104° and a roseolous rash with general enlargement of the lymphatic glands developed. These symptoms gradually disappeared, but after a sub-febrile period the temperature again rose and on April 18th an ulcer appeared on the alveolar margin of the gum and was followed by the formation of a submaxillary abscess. This was opened, but in spite of various dressings the wound would not heal and local treatment of the ulcer was equally unsuccessful. On two occasions during the course of the illness (in addition to the one mentioned above) the roseolous rash returned and was followed by a fine desquamation of the cuticle. About the middle of June the patient developed signs of intra-cranial mischief, and in the course of a few days became comatose and died. At the post-mortem examination (the brain only being examined) thrombosis of the cerebral sinuses was observed. A culture obtained from the ulcer showed *Staphylococcus pyogenes aureus*. The case illustrates the diminished resisting power capable of being produced by an attack of measles. The roseolous rash and other symptoms were doubtless all due to general infection.

The second case (Dr Anderson Smith)[2] suggests that the toxins associated with the infective agent in measles may in some instances have a serious effect on the central nervous system. The patient was a girl of five years. When suffering from a fairly severe attack of measles she developed severe cerebral symptoms including vomiting, squinting, retraction of the head, and unconsciousness. After a few days the more acute symptoms subsided, but the patient was found to be aphasic and there was pronounced wasting. At the end of a month aphasia still persisted and there was some rigidity of the limbs with ankle-clonus and increase of the knee-jerks. More recently there were evidences of improvement both as regards speech and motor power. Another feature of the illness was general cutaneous anæsthesia preceded apparently by hyperæsthesia. At no time was there optic neuritis or discharge from the ear. The patient was shown to the Society for the Study of Disease in Children and the general opinion was in favour of the view that the condition was a toxæmic one, and that in view of the evidences of improvement an advance towards a complete convalescence might be expected.

In a third case (Mr. Arnold Lawson)[3] a lad of nine years, some six weeks after an attack of measles, developed an internal squint due to a paralysis of the external rectus muscle. It is suggested that the paralysis is comparable to similar conditions occasionally seen after the exanthemata and diphthena.

[1] Brit. Jour. Child. Dis., March 1904. [2] Ibid., June 1904. [3] The Polyclinic, March 1904.

